# Effect of lipid-lowering medications in patients with coronary artery bypass grafting surgery outcomes

**DOI:** 10.1186/s12871-022-01675-9

**Published:** 2022-04-26

**Authors:** Chunxia Shi, Zugui Zhang, Jordan Goldhammer, David Li, Bob Kiaii, Victor Rudriguez, Douglas Boyd, David Lubarsky, Richard Applegate, Hong Liu

**Affiliations:** 1 Department of Anesthesiology, Peking University International Hospital, Beijing, China; 2 Department of Anesthesiology and Pain Medicine, University of California Davis Health, 4150 V Street, Suite 1200, Sacramento, CA 95817 USA; 3 Institute for Research On Equality and Community Health, Christiana Care, Newark, DE USA; 4 Department of Anesthesiology, Sidney Kimmel Medical College at Thomas Jefferson University Hospital, Philadelphia, PA 19107 USA; 5 Department of Surgery, University of California Davis Health, Sacramento, CA USA

**Keywords:** CABG, CPB, Perioperative lipid-lowering drug, Adverse events, Outcome

## Abstract

**Background:**

Increased life expectancy and improved medical technology allow increasing numbers of elderly patients to undergo cardiac surgery. Elderly patients may be at greater risk of postoperative morbidity and mortality. Complications can lead to worsened quality of life, shortened life expectancy and higher healthcare costs. Reducing perioperative complications, especially severe adverse events, is key to improving outcomes in patients undergoing cardiac surgery. The objective of this study is to determine whether perioperative lipid-lowering medication use is associated with a reduced risk of complications and mortality after coronary artery bypass grafting (CABG) with cardiopulmonary bypass (CPB).

**Methods:**

After IRB approval, we reviewed charts of 9,518 patients who underwent cardiac surgery with CPB at three medical centers between July 2001 and June 2015. The relationship between perioperative lipid-lowering treatment and postoperative outcome was investigated. 3,988 patients who underwent CABG met inclusion criteria and were analyzed. Patients were divided into lipid-lowering or non-lipid-lowering treatment groups.

**Results:**

A total of 3,988 patients were included in the final analysis. Compared to the patients without lipid-lowering medications, the patients with lipid-lowering medications had lower postoperative neurologic complications and overall mortality (*P* < 0.05). Propensity weighted risk-adjustment showed that lipid-lowering medication reduced in-hospital total complications (odds ratio (OR) = 0.856; 95% CI 0.781–0.938; *P* < 0.001); all neurologic complications (OR = 0.572; 95% CI 0.441–0.739; *P* < 0.001) including stroke (OR = 0.481; 95% CI 0.349–0.654; *P* < 0.001); in-hospital mortality (OR = 0.616; 95% CI 0.432–0.869; *P* = 0.006; *P* < 0.001); and overall mortality (OR = 0.723; 95% CI 0.634–0.824; *P* < 0.001). In addition, the results indicated postoperative lipid-lowering medication use was associated with improved long-term survival in this patient population.

**Conclusions:**

Perioperative lipid-lowering medication use was associated with significantly reduced postoperative adverse events and improved overall outcome in elderly patients undergoing CABG surgery with CPB.

## Introduction

With increased life expectancy and improved medical technology, more elderly patients are receiving cardiac surgery that undoubtedly carries an increased risk of postoperative morbidity and mortality. It has been shown that the overall complication rate of cardiac surgery may be as high as 66% [1, 2]. These complications can lead to decreased quality of life, shortened life expectancy and higher healthcare costs. Reducing perioperative complications, especially severe adverse events, is key to improving outcomes in patients undergoing cardiac surgery.

Statins are inhibitors of the enzyme 3-hydroxy-3-methylglutaryl-coenzyme A reductase and are one of the most important lipid-lowering medicines. Their use is well established in treating hyperlipidemia by lowering plasma low-density lipid cholesterol (LDL-C) level [3]. Apart from their lipid-lowering ability, the pleiotropic effect of statins has received more attention in recent years. The pleiotropic effect is associated with the improvement of endothelial function, reduction of vascular inflammation, stabilization of atherosclerotic plaques, inhibition of oxidative stress and prevention of vascular remodeling [4–7]. Because of these benefits, statins have been widely used for patients who suffer from cardiovascular diseases and undergo cardiac surgery. Studies have shown that perioperative statin therapy is associated with reductions of atrial fibrillation (AF), myocardial infarction (MI), vascular thrombosis, acute kidney injury (AKI), and infection after cardiac surgery [8–13]. Statin use has also been shown to reduce mortality and shorten the length of hospital stay (LOS) in the same patient population [14–16]. It has been suggested that surgical patients should resume statin therapy in the perioperative period, particularly patients with atherosclerotic cardiovascular disease [17]. The goal of this study was to evaluate the effects of perioperative lipid-lowering medications on the incidence of postoperative adverse events and improving both short- and long-term outcomes in patients undergoing CABG/CABG with other procedures.

## Methods

This retrospective study consisted of 9,518 patients who underwent various types of cardiac surgery from July 2001 to June 2015 at three different US medical centers. The study was approved by the IRB committees from University of California Davis, Jefferson University and Christiana Care and consent was waived because this is a retrospective data analysis. All the methods were performed in accordance with the Declaration of Helsinki. After IRB approval, the patient’s demographic information and perioperative data were reviewed from the institutional Society of Thoracic Surgeons (STS) database and there were no humans participated in this study directly. The perioperative period is the time the patient goes into the hospital, clinic, or doctor's office for surgery until the time the patient is discharged home. Inclusion criteria were patients who underwent CABG or CABG with other procedures. Exclusion criteria were patients who underwent cardiac surgery without CPB, any type of transplantation procedure, aortic procedure, minimally invasive surgery, non-CABG, and emergency surgery. The clinical follow-up was up to November 26, 2015. Patients with CABG surgery were identified and divided into two groups according to whether lipid-lowering medications were used before the surgery: the lipid-lowering treatment (LLT) group and non-lipid-lowering treatment (non-LLT) group. Postoperative complications, major adverse events, and mortality were evaluated. Postoperative lipid-lowering medication use was also recorded.

### Statistical analysis

Continuous variables were reported as mean ± standard deviation [18] and compared with a 2-sample t tests if normally distributed or as median (interquartile) and compared with Wilcoxon rank test if necessary. Categorical variables were reported as number and percentage, and compared with χ^2^ test or fisher’s exact test if necessary. Univariate and multivariate logistic regression were performed to assess associations between lipid-lowering use and demographic and clinical outcome variables. The inverse probability of treatment weighting (IPTW) method was used in logistic regression model to evaluate the lipid-lowering medication effect on the study patients for all patients and for patients undergoing CABG without other procedures. The propensity score with the nearest method was used in survival analysis to compare the results of time-event endpoints between groups based on lipid-lowering medication use. Data management and statistical analyses were conducted with R software (version 3.5.2). The Kaplan–Meier method was applied to study patient survival. The log-rank test was used to ascertain differences between groups. *P* value of equal or less than 0.05 was considered statistically significant.

## Results

**Fig. 1:**
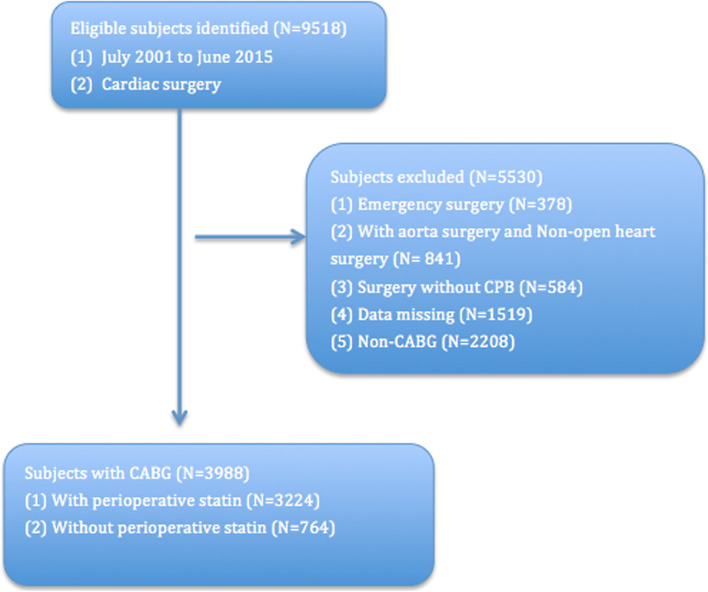
Recruiting of study sample. CABG = coronary artery bypass; CPB = cardiopulmonary bypass

Data from a total of the 9,518 patients were reviewed and 5,530 cases were excluded for not meeting the inclusion criteria. A total of 3,988 patients were included in the final analysis (Fig. 1). A higher proportion of LLT patients also had diabetes, hypertension, smoking, peripheral arterial disease, cerebrovascular disease, previous MI and previous cardiac surgery. Patients in the LLT group had a higher body mass index (BMI), more underwent CABG with other procedures and were more likely to be female. More patients in the non-LLT group had history of cardiogenic shock compared to those in the LLT group. Before surgery, more patients in LLT group received angiotensin converting enzyme inhibitors (ACEI), angiotensin receptor blocker (ARB), aspirin and βblocker treatment. The average age was more than 65 years old in both groups and did not differ between groups. Demographic and clinical characteristics are summarized in Table 1.

**Table 1:** Demographics and clinical characteristics of study cohort

Characteristics	Non-LLT Group	LLT Group	*p*-value	PS adjusted Non-LLT	PS adjusted LLT	*p*-value
Age(years), Mean [SD]	65.5(12.1)	66.4(10.7)	0.231	66.4(11.3)	66.0(10.8)	0.394
Female, N (%)	225(29.5)	778(24.1)	0.003	1060(26.1)	1002(25.2)	0.637
BMI, Mean [SD]	28.7(8.4)	29.5(7.5)	0.007	29.1(5.9)	29.2(5.9)	0.778
Diabetes, N (%)	231(30.2)	1361(42.2)	< 0.001	1675(41.3)	1593(40.0)	0.591
Renal Failure, N (%)	47(6.2)	169(5.2)	0.363	211(5.2)	214(5.4)	0.861
Dialysis, N (%)	32(4.2)	120(3.7)	0.617	151(3.7)	151(3.8)	0.943
Hypertension, N (%)	556(72.8)	2755(85.5)	< 0.001	3378(83.3)	3308(83.1)	0.896
Smoking, N (%)	359(47.0)	1645(51.0)	0.049	2025(49.9)	1993(50.0)	0.957
Chronic lung disease, N (%)	132(17.3)	532(16.5)	0.643	631(15.6)	660(16.6)	0.539
Peripheral arterial disease, N (%)	87(11.4)	497(15.4)	0.006	636(15.7)	584(14.7)	0.593
Cerebrovascular disease, N (%)	91(11.9)	587(18.2)	< 0.001	720(17.8)	678(17.0)	0.717
Creatinine, Mean [SD]	1.26(1.16)	1.23(1.04)	0.523	1.24(1.06)	1.23(1.07)	0.885
Previous cardiac surgery, N (%)	17(2.2)	139(4.3)	0.010	177(4.4)	157(3.9)	0.718
Previous MI, N (%)	253(33.1)	1482(46.0)	< 0.001	1763(43.4)	1728(43.4)	0.985
Previous heart failure, N (%)	206(27.0)	811(25.2)	0.325	1007(24.8)	1009(25.3)	0.792
Cardiogenic shock, N (%)	25(3.3)	51(1.6)	0.003	75(1.8)	75(1.9)	0.942
ACEI. ARB treatment, N (%)	297(38.9)	1568(48.6)	< 0.001	1986(48.9)	1872(47.0)	0.410
Aspirin treatment, N (%)	547(71.6)	2853(88.5)	< 0.001	3500(86.3)	3403(85.5)	0.519
β-blocker treatment, N (%)	485(63.5)	2573(79.8)	< 0.001	3136(77.3)	3058(76.8)	0.776
EF < , Mean [SD]	50.2(14.49)	51.03(13.62)	0.132	51.1(13.3)	50.9(13.8)	0.719
Valve procedure, N (%)	239(31.3)	705(21.9)	< 0.001	911(22.4)	938.2(23.6)	0.536
Other cardiac surgery, N (%)	85(11.1)	273(8.5)	0.025	325(8.0)	353(8.9)	0.449
CPB time, Mean [SD]	136.7(78.9)	132.0(71.4)	0.109	134.2(76.6)	132.7(72.1)	0.685
Clamp time, Mean [SD]	97.6(61.8)	92.7(54.5)	0.028	94.8(61.2)	93.2(54.9)	0.583

*LLT* lipid lowing treatment, *N* numbers, *SD* standard deviation, *BMI* body mass index, *MI* myocardial infarction, *EF* ejection fraction, *CPB* cardiopulmonary bypass, *DC-lipid lowering* discharge with lipid lowering medications, *PS* propensity score

As shown in Table 2, the patients in LLT group had a significantly lower incidence of in-hospital complications including neurologic complications and shorter ICU LOS compared to patients in non-LLT group (*P* < 0.05). The overall mortalities were significant lower in LLT group (*P* < 0.05). There was no difference in postoperative infection, prolonged ventilation, renal failure and requirement of renal replacement therapy, new onset atrial fibrillation (AF) or LOS between the groups. After propensity score adjustment, the incidence of postoperative stroke, other neurological complications (transient ischemic attack, encephalopathy, coma) and overall mortality in the LLT group were still significantly lower than those in non-LLT group.

**Table 2:** Effect of lipid-lowering medications on outcomes

Outcome	Non-LLT Group	LLT Group	*P* value	PS adjusted Non-LLT	PS adjusted LLT	*P* value
**In-hospital complications, N (%)**	300(39.3)	1086(33.7)	0.004	1508.9(37.2)	1339.8(33.6)	0.109
**Infection, N (%)**	8(0.9)	40(1.2)	0.493	38.3(0.9)	45.9(1.2)	0.639
**Stroke, N (%)**	21(2.7)	48(1.5)	0.025	123.9(3.1)	59.4(1.5)	0.016
**Other neurologic complications, N (%)**	29(3.8)	76(2.4)	0.035	163.1(4.0)	93.3(2.3)	0.033
**Ventilation prolong, N (%)**	129(16.9)	453(14.1)	0.053	623.7(15.4)	567.5(14.3)	0.495
**Renal failure, N (%)**	29(3.8)	133(4.1)	0.754	169.9(4.2)	165.1(4.1)	0.964
**Renal dialysis, N (%)**	11(1.4)	64(2.0)	0.396	65.5(1.6)	79.8(2.0)	0.563
**New onset AF, N (%)**	192(25.1)	797(24.7)	0.850	979.9(24.2)	979.4(24.6)	0.824
**Total ICU hours, Mean **[17]	121.9(343.1)	102.6(156.2)	0.020	116.7(305.3)	103.7(158.2)	0.685
**Length of hospital stay, Mean **[17]	9.30(8.49)	9.39(35.73)	0.947	8.87(7.66)	9.36(33.24)	0.430
**In-hospital Mortality, N (%)**	13(1.7)	39(1.2)	0.368	84.5(2.1)	51.1(1.3)	0.213
**30d Mortality, N (%)**	15(2.0)	49(1.5)	0.473	79.8(2.0)	62.1(1.6)	0.539
**Overall Mortality, N (%)**	111(14.5)	361(11.2)	0.012	606.2(14.9)	448.9(11.3)	0.017

*LLT* lipid lowing treatment, *N* numbers, *SD* standard deviation, *AF* atrial fibrillation, *PS* propensity score

To determine the effect of lipid-lowering medications on the outcomes of CABG surgery only, a multivariate logistic regression analysis, with IPTW adjustment model (Table 3) was performed. This showed the LLT group had fewer in-hospital complications (OR = 0.856; 95% CI 0.781–0.938; *P* < 0.001); less in-hospital mortality (OR 0.616; 95% CI 0.432–0.869; *P* = 0.006); lower overall mortality (OR = 0.723; 95% CI 0.634–0.824; *P* < 0.001); and fewer neurologic complications (OR = 0.572; 95% CI 0.441–0.739; *P* < 0.001). Postoperative stroke, a devastating complication, was also lower in the LLT group (OR = 0.481; 95% CI 0.349–0.654; *P* < 0.001).

**Table 3:** Association between lipid-lowering treatment and postoperative complication and mortality after propensity weighted risk-adjustment

Outcomes	OR	Coefficient	95%CI	*P* value
In-hospital complication	0.856		0.781–0.938	< 0.001
Infection	1.223		0.795–1.890	0.361
Stroke	0.481		0.349–0.654	< 0.001
Neurologic complication	0.572		0.441–0.739	< 0.001
Ventilation prolonged	0.915		0.809–1.-35	0.156
Renal failure	0.989		0.795–1.232	0.922
Renal dialysis	1.245		0.896–1.736	0.192
New onset AF	1.024		0.925–1.134	0.645
In-hospital Mortality	0.616		0.432–0.869	0.006
30-day Mortality	0.789		0.563–1.101	0.165
Overall Mortality	0.723		0.634–0.824	< 0.001
Total ICU hours		-12.985	-28.120–2.150	0.093
Length of hospital stay		0.491	-1.000–1.983	0.518

*OR* odds ratio, *AF* atrial fibrillation, *ICU* intensive care unit, CI confidence interval

**Fig. 2:**
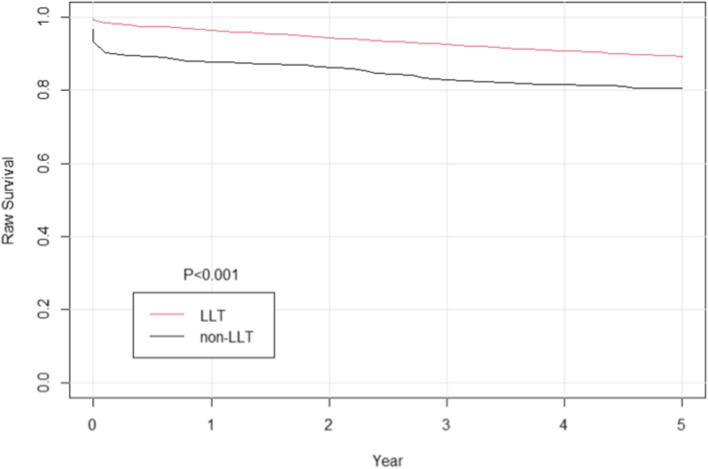
Cumulative event curve for all-cause mortality. The grey line represents discharge without lipid-lowering treatment (LLT); and red line represents discharge with lipid-lowering treatment

**Fig. 3:**
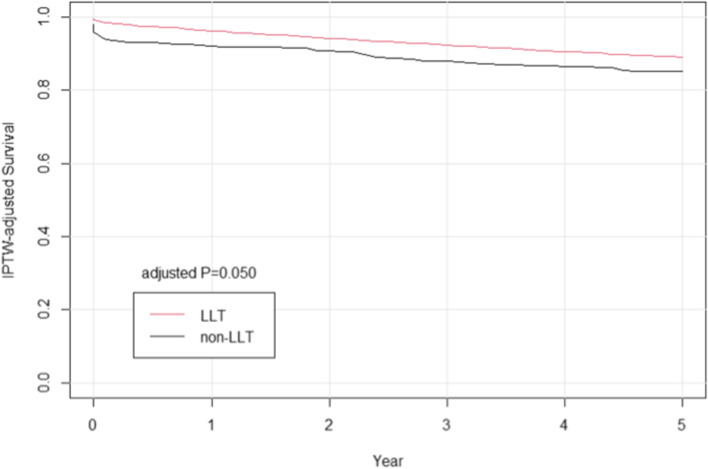
Cumulative event curve for all-cause mortality after inverse probability of treatment weighting (IPTW) adjusted method. The grey line represents discharge without lipid-lowering treatment (LLT); and red line represents discharge with lipid-lowering treatment

Survival probability was calculated with Kaplan–Meier methods and compared with the use of a log-rank test after propensity score. In this study, postoperative lipid-lowering medication use was shown to be associated with improved long-term survival more than 5 years in patients after cardiac surgery (Figs. 2 and 3).

## Discussion

This study demonstrated that preoperative lipid-lowering medication use was associated with decreased overall in-hospital complications especially neurologic complications, and lower mortality in elderly patients undergoing CABG with CPB. Postoperative lipid-lowering medication use was shown to be associated with improved long-term survival more than 5 years in the same patient population.

Statins are one of the most important therapies for patients with atherosclerotic coronary artery disease [19]. In this study, 97.2% of the patients receiving lipid-lowering medications were receiving statins. Previous studies have reported that statin pretreatment in patients undergoing CABG could reduce postoperative mortality, MI, stroke, AF, and length of hospital stay [20]. A multicenter prospective study of 5,436 patients undergoing CABG surgery indicated that perioperative statin therapy was independently associated with a significant reduction in the risk of early cardiac death after primary elective CABG surgery [21]. Another study of patients undergoing CABG with CPB also provided evidence that preoperative statin therapy reduces the risk of early mortality [22]. However, most of these studies only included the patients undergoing isolated CABG surgery. In order to assess the impact of lipid-lowering medications on a broader patient population, our study included both isolated CABG surgery and also CABG combined with other cardiac procedures. We found that neurologic complications (including stroke), in-hospital mortality and overall mortality were all reduced significantly in the patients treated with lipid-lowering medications.

In studies examining statin therapy and postoperative outcome in patients who underwent non-emergent valve surgery, the authors found that preoperative statin therapy was associated with significantly reduced mortality [23, 24]. We found statins were associated with reduced postoperative complications in patients undergoing CABG with or without other procedures suggesting that the beneficial effects from the combination of lipid-lowering, pleiotropic, anti-inflammatory, immunomodulatory, and antioxidant properties [25–27] can benefit patients undergoing combined procedures as well. Statins inhibit the production of several proinflammatory cytokines, including tumor necrosis factor alpha (TNF-α), Interleukin-6 (IL-6), and IL-8, resulting in the reduction of C-reactive protein levels and suppression of natural killer cells [28–30]. A meta-analysis including 10 cohort studies with a total of 147,263 participants showed that statin use was associated with a lower incidence of postoperative infectious complications in both cardiac and non-cardiac surgery possibly due to anti-inflammatory or immunomodulatory properties [10]. Another study showed that male gender, increased BMI, presence of left main coronary artery disease, and increased LOS were significantly associated with the risk of deep sternal wound infections, while the presence of hypercholesterolemia was a protective factor hypothesized to be due to long-term statin use [31]. Although studies have demonstrated statin therapy was associated with a reduced risk of postoperative infection [10, 31, 32], other studies including the present one did not support this finding [33, 34].

Similarly, there is a high incidence of AKI after cardiac surgery which is associated with increased postoperative mortality and hospital costs [35]. The effect of statin on AKI and perioperative renal replacement therapy is another controversial topic. Some clinical studies have shown positive renal protection in patients using statins undergoing cardiac surgery [13, 36, 37]. However, other studies did not reach the same conclusion [20, 38–40]. Our study found perioperative statin therapy was not associated with a reduced incidence of cardiac surgery-associated AKI.

Postoperative atrial fibrillation (POAF) is one the most frequent complications after cardiac surgery, and the role of statin use in preventing POAF has been extensively studied. POAF is triggered by the interaction of different pathogenic factors and inflammatory processes. Sulzgruber and colleagues suggested that preoperative statin therapy decreased the development of POAF due to anti-inflammatory properties, such as reducing the level of C-reactive protein (CRP) [41]. It has also been suggested that statin therapy has an overall protective effect against POAF especially in patients undergoing CABG [9]. We did not find statin therapy is protective against POAF in this study.

Postoperative stroke in cardiac surgery patients increases morbidity and mortality [42, 43]. Evidence has shown that statins can exert multidirectional effects, interfering with reactive oxygen species development, clot formation, endothelial function, and brain plasticity which might play an essential role in the treatment of ischemic stroke, preventing stroke recurrence and cardiovascular events, and improving functional performance [44]. Another study also confirmed the effect of statin-based therapy for primary and secondary prevention of ischemic stroke [45]. Our results show preoperative lipid-lowering treatment was associated with reduced postoperative stroke and other neurologic complications which supports a neuroprotective effect of statins following CABG.

The results from the present study show that patients on lipid-lowering medications postoperatively had a longer postoperative survival time, suggesting that lipid-lowering medications could reduce postoperative mortality after cardiac surgery for one year or longer. This is consistent with findings shown by others and it is suggested that the anti-inflammatory effect of statin could be one of the mechanisms [46–48]. Another study in patients with atherosclerotic cardiovascular disease evaluated major adverse cardiac events (MACE) defined as MI, angina, coronary revascularization, ischemic stroke, transient ischemic attack (TIA), or peripheral artery disease. The results indicated that statin use within 30 days of hospital discharge was associated with a lower risk of MACE [49].

This study has several limitations. First, it is a retrospective, nonrandomized study with potential hidden biases that could have affected our analysis. Second, we knew whether patients were receiving perioperative statin treatment, but we were unable to classify the types, doses, and timing of statin therapy. Lastly, compared to non-LLT patients, patients with lipid-lowering therapy were more likely to be concomitantly treated with aspirin, β-blockers, ACEI or ARB which could have beneficial effects on postoperative outcomes [50], but were also more likely to have comorbid conditions that could have negative effects on postoperative outcomes. Although we have taken these factors into account in our statistical analysis, they may still have confounding effects.

In conclusion, among patients undergoing CABG with CPB, perioperative statin use was associated with reduction of postoperative complications and mortality. Postoperative lipid-lowering medication use was shown to be associated with improved 5-year long-term survival. Our study suggests that patients undergoing CABG should consider starting statin therapy perioperatively unless contraindicated, especially for those with significant risk factors such as atherosclerotic cardiovascular disease.

## Data Availability

The datasets used and/or analyzed during the current study are available from the corresponding author on reasonable request.
